# Advanced Mechanical Testing Technologies at the Cellular Level: The Mechanisms and Application in Tissue Engineering

**DOI:** 10.3390/polym15153255

**Published:** 2023-07-31

**Authors:** Yingxuan Zhu, Mengqi Zhang, Qingqing Sun, Xiaofeng Wang, Xiaomeng Li, Qian Li

**Affiliations:** 1 School of Mechanics and Safety Engineering, Zhengzhou University, Zhengzhou 450001, China; 2 National Center for International Joint Research of Micro-nano Moulding Technology, Zhengzhou University, Zhengzhou 450001, China; 3 School of Materials Science and Engineering, Zhengzhou University, Zhengzhou 450001, China

**Keywords:** mechanical testing technologies, tissue engineering scaffolds, mechanical microenvironment, biomechanics, hydrogel

## Abstract

Mechanics, as a key physical factor which affects cell function and tissue regeneration, is attracting the attention of researchers in the fields of biomaterials, biomechanics, and tissue engineering. The macroscopic mechanical properties of tissue engineering scaffolds have been studied and optimized based on different applications. However, the mechanical properties of the overall scaffold materials are not enough to reveal the mechanical mechanism of the cell–matrix interaction. Hence, the mechanical detection of cell mechanics and cellular-scale microenvironments has become crucial for unraveling the mechanisms which underly cell activities and which are affected by physical factors. This review mainly focuses on the advanced technologies and applications of cell-scale mechanical detection. It summarizes the techniques used in micromechanical performance analysis, including atomic force microscope (AFM), optical tweezer (OT), magnetic tweezer (MT), and traction force microscope (TFM), and analyzes their testing mechanisms. In addition, the application of mechanical testing techniques to cell mechanics and tissue engineering scaffolds, such as hydrogels and porous scaffolds, is summarized and discussed. Finally, it highlights the challenges and prospects of this field. This review is believed to provide valuable insights into micromechanics in tissue engineering.

## 1. Introduction

Biophysical signals, including mechanical ones, have attracted a lot of investigation in recent years. The general mechanical characteristics of hydrogels, such as stiffness [1,2] and viscoelasticity [3,4,5], have frequently been used as biomechanical cues in previous research on cell cultivation to influence cellular activities like adhesion, spreading, migration, proliferation, and differentiation. However, native tissues frequently exhibit non-uniform and heterogeneous structural and mechanical characteristics [6]. Mechanics at the cellular scale, rather than those at a more macroscopic level, govern the biophysical interplay between cells and their surroundings. For example, mechanical measurements at the cellular level have revealed that the pericellular matrix (PCM) surrounding chondrocytes has an elastic modulus in the tens of kilopascals, while the extracellular matrix (ECM) of cartilage can reach several hundred kilopascals [7,8]. When exposed to external loads, the PCM undergoes significant strain that varies with the depth of the area which it occupies [9,10]. The substantial significance of PCM in controlling the mechanical environment of chondrocytes is revealed by the complicated and non-uniform mechanical environment within articular cartilage cells. Experimental and mechanical modeling investigations of the “chondron”, which consists of the PCM and chondrocyte, have shown that the structure and characteristics of the PCM have the function to protect chondrocytes by tuning their mechanical microenvironment [11,12].

Similarly, the macroscopic mechanical properties of scaffolds cannot accurately represent the mechanical microenvironment experienced by cells. It is the local microscale mechanics that have direct impacts on cell behavior and functionality in tissue engineering scaffolds. For example, in 3D-printed PCL scaffolds filled with cell-laden hydrogels, the mechanical microenvironment of the cells is provided by the local hydrogel, and the overall mechanical properties of the scaffold have little impact on the behavior and fate of individual cells [13]. In addition, cellular mechanics is a cutting-edge field involving the investigation of the elastic modulus, viscoelasticity, membrane tension, and cell-matrix traction forces [14,15,16]. These mechanical properties have various effects on cell migration [17], proliferation [18], and differentiation [19,20]. For example, studying cell membrane tension or the modulus of the cell membrane/cytoskeleton can help to reveal how mechanotransduction affects cell secretion and differentiation. Furthermore, changes in the mechanical properties of cells are associated with disease progression. Cancer cells have a different rheological viscoelasticity from normal cells [21], and erythrocytes infected with malaria parasites exhibit considerable deformation changes under tensile optical stress compared to healthy erythrocytes [22].

Therefore, it is of great significance and there is an urgent demand to detect the local mechanical microenvironment around cells, as well as the mechanics of the cell itself, and to explore the role and mechanism of those mechanics [23,24]. Cells are continuously and dynamically subjected to mechanical forces through cell–cell and cell–matrix interactions. Due to mechanical strain between adjacent cells, these stresses activate mechanosensitive ion channels, redistribute adhesion ligands, and initiate cell–cell junctional responses, or generate diverse biological signaling through mechanotransduction [25,26,27,28]. Therefore, quantitative micromechanical assessment of the microenvironment at the cellular scale is crucial for studying cell development and morphogenesis [29]. To this end, Young’s modulus (E) and Poisson’s ratio (μ) are fundamental mechanical properties of materials and are often used to define material properties for mechanical microenvironments. They reflect the stiffness and elasticity of the ECM and control the magnitude and distribution of stress (σ) and strain (ε) in response to external mechanical stimuli.

There have been many reviews on mechanical transduction and the mechanics of cell–microenvironment interaction [6,23,30,31,32]. This review will primarily focus on advanced detection approaches as well as how to examine cell-scale mechanics in scaffold material systems of various dimensions and architectures. In the first section, we will introduce and explain the mechanical principles of tools utilized in quantitative biomechanics, such as atomic force microscopes (AFM), optical tweezers (OT), magnetic tweezers (MT), and traction force microscopes (TFM). In the second part, the detection of forces created by cells or sensed by cells in artificial extracellular matrices will be emphasized. Existing methods for monitoring cell mechanical stresses will be discussed, as well as how they are used to analyze the mechanical mediation effect of biomimetic scaffolds such as hydrogels and porous scaffolds. This review aims to shed light on the fields of biomechanics and mechanics of materials for tissue engineering. The discussions and conclusions presented in this paper may serve to guide future research works.

## 2. Advanced Mechanical Testing Technologies and Their Mechanisms

**Figure 1:**
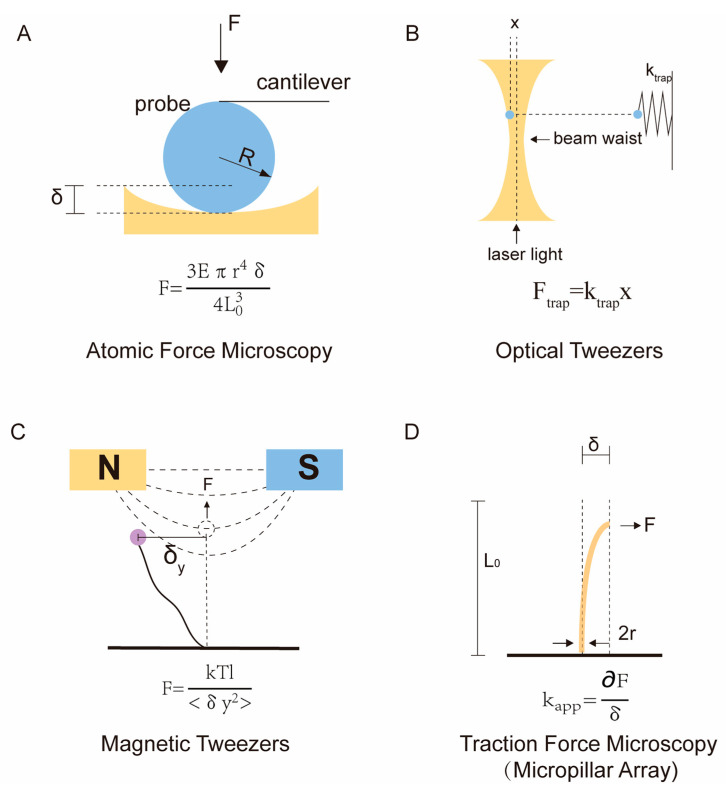
Schematic diagram and mechanical principles of testing technologies. (**A**) Contact model of atomic force microscopy spherical probe with biological samples. (**B**) Calculating optical tweezer force based on Hooke’s law. (**C**) Calculating magnetic tweezer force based on particle displacement amplitude $\;\langle \delta y^{2}\rangle$. (**D**) Schematic diagram of micropillar array based on the principles of traction force microscopy. The cellular traction force causes deflection at the top of the cantilever beam.

Single-molecule force spectroscopy techniques, optical tweezers (OT), magnetic tweezers (MT), and atomic force microscopy (AFM) are used to explore the unknown mechanical properties of cells or their matrices to obtain their modulus (Figure 1) [33]. Among the mechanical detection technologies, traction force microscopy (TFM) is utilized in existing cell mechanics research methods to detect the traction forces imposed by cells on the matrix, and requires researchers to have prior knowledge of the elastic mechanical properties of the matrix [34,35], including the E and μ. The mechanical stimulus, as one of the major physical signals that regulate cell fate and tissue development, was rarely examined quantitatively until the aforementioned instruments were established and improved in recent decades. The most regularly used single-molecule force spectroscopy techniques for examining the forces and movements of biomolecules are OT, MT, and AFM. OT and MT, for example, can be used to measure force at the molecular scale of subcellular structures such as DNA, RNA, proteins, and enzymes [36,37,38]. In this review, we focus only on their applications at the single-cell level rather than at the subcellular molecular scale. In addition, finite element analysis (FEA) is widely used in cell mechanics. Since it is a numerical analysis technique for simulating mechanical behavior, it is subject to fewer technical restrictions [39].

### 2.1. Atomic Force Microscopy

**Figure 2:**
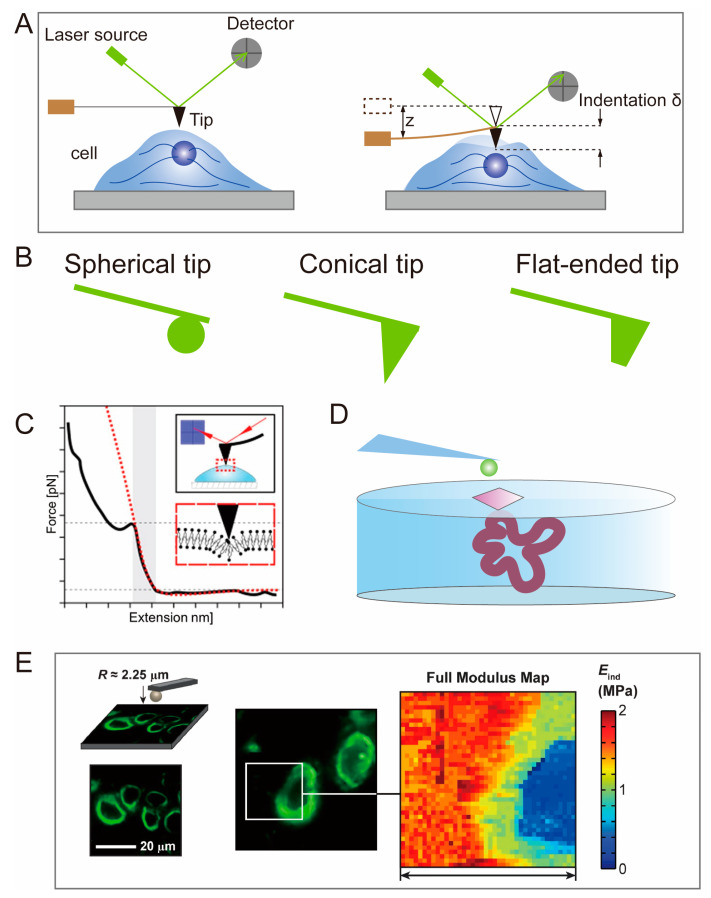
The mechanical detection using AFM. (**A**) Schematic diagram of cell device for AFM detection of two-dimensional culture. (**B**) Schematic of different shapes of probe tips. (**C**) Schematic of the AFM cell membrane indentation for the Young’s Modulus measurements [49]. Copyright 2022, American Chemical Society. (**D**) Schematic of AFM-based stiffness mapping strategy of organoid encapsulated in hydrogel. (**E**) Representative indentation modulus map with corresponding collagen VI IF image [50]. Copyright 2020, Elsevier.

AFM is used to test the surface topography and modulus of cells or tissue engineering scaffolds. Binnig designed AFM in 1985 to address the limitations of scanning tunneling microscopy (STM) in measuring live cells [40,41], which was then further improved by Binnig, Christoph Gerber, and Calvin Quate. Samples can be placed in a variety of environments, including standard atmospheric conditions or liquids, enabling the monitoring of biological samples and their dynamic processes in liquid environments. A vibrating cantilever with a conical or spherical tip mounted on the end is driven by a piezoelectric piece of ceramic and compresses the sample along the Z-axis. Force curves are obtained by lowering the position of the probe, in the AFM method, until the tip touches the biological sample. If this action continues, the tip will cause deformation or indentation of the sample until a trigger threshold for probe position or cantilever deformation is reached (Figure 2A). Then, the probe is lifted until the probe tip is no longer in contact with the sample. The measured probe deformation and Z sensor position information during this load–unload cycle can be converted into a force–distance curve. By fitting the slope of the force–distance curve using a model in the AFM software, the modulus of the biological sample can be accurately assessed [42,43] and adhesive forces or adhesion energies can be calculated from the retraction curves [44,45]. The imaging of ideal linear elastic samples is achieved by collecting force–distance curves of discrete points or continuous regions on the sample surface using “force mapping.” In force mapping, force curves are continually recorded while laterally scanning the tip on the sample, enabling quantitative assessment of the spatial distribution of the sample’s elastic modulus [46,47,48].

The Hertz/Sneddon model is currently the most commonly used model in AFM measurements [51,52]. This model assumes a linear elastic relationship between the tip and the sample, neglecting the effects of adhesion or other surface interactions. The original Hertz model was applicable only to spherical tips, but Sneddon later extended it to include other axisymmetric geometries [53]. Researchers can select the shape of the tip in the AFM software, including spherical, conical, and flat-ended tips (Figure 2B). For biological samples, a spherical probe is commonly used (Figure 1A), and the corresponding Hertz contact model is given by:

(1) $$F=E_{eff}\cdot [\left(a^{2}+R_{P}^{2}\right)\cdot ln\left(\frac{R_{P}+a}{R_{P}-a}\right)-2aR_{P}],$$

(2) $$\delta =\frac{a}{2}ln\frac{R_{P}+a}{R_{P}-a}$$

where $E_{eff}$ is the Young’s modulus, a is the contact radius, $R_{P}$ is the radius of the indenter, and *δ* is the indentation depth.

It is important to note that, when applying the Hertz/Sneddon model, the indentation depth should be less than 10% of the sample thickness to avoid the influence of the underlying substrate [48].

AFM has helped in investigating the microscale mechanical performance of tissues and the ECM at the cellular level due to its ability to operate in liquid settings and its high resolution of up to 1 nm. Although larger spherical probes are usually utilized for biological samples [54], AFM’s resolution can still exceed 0.1 μm, allowing for the assessment of plasma membranes (PM) or cellular cytoskeleton mechanical characteristics, rather than just the cell as a whole (Figure 2C). For example, Darius Z Lachowski et al. investigated the impact of membrane tension on vesicle transport in cells [49]. By limiting the approach velocity of the cantilever (2 μm/s) and the voltage setpoint (0.1 V) to ensure that the indentation curves corresponded to the PM, the membrane stiffness of hematopoietic stem cells (HSCs) cultured on hydrogels was found to range from 1.0 kPa to 3.6 kPa. M Cascione et al. utilized AFM to evaluate the different Young’s moduli of a cell nucleus and cytoplasm [55,56]. These results confirm the effectiveness of AFM in probing the mechanical properties of the cellular microenvironment.

AFM can also assess the Young’s modulus of cell-loaded 3D hydrogels. There is an interaction between cells and hydrogels, and cells can actively remodel the mechanical microenvironment around them by degrading or depositing hydrogels [57,58]. By probing the modulus of the hydrogel surrounding the cells using AFM, a clear difference in stiffness between cells, PCM, and bulk hydrogels can be found (Figure 2D) [59,60]. However, there are limitations in the single-use application of AFM in practical biomedicine [61]. AFM probes require continuous measurements in different regions and cannot directly detect specific regions of interest, such as the PCM and ECM [62]. This limitation can be addressed by tracking the locations of specific proteins on or within cells in fluorescence imaging using confocal laser scanning microscopy (CLSM) (Figure 2E) [50,63]. 

### 2.2. Optical Tweezer and Magnetic Tweezer

**Figure 3:**
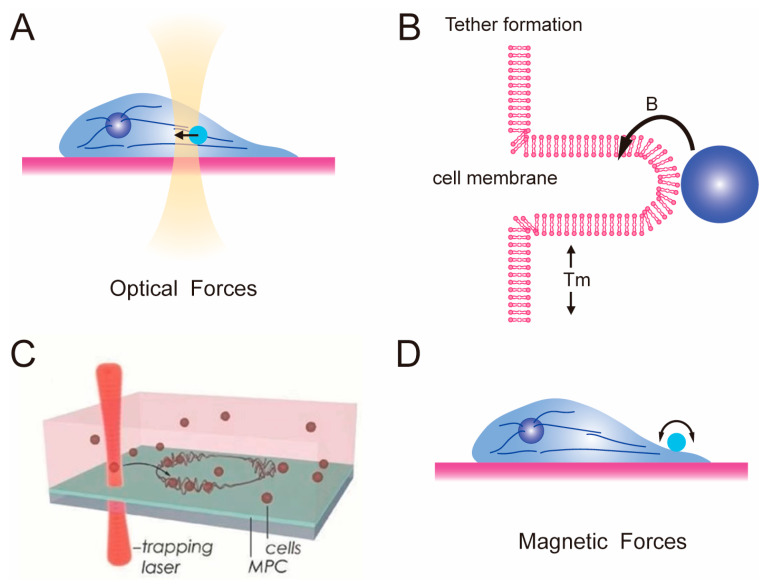
Schematic diagram of the operating principles of OT and MT. (**A**) Schematic diagram of optical tweezer. (**B**) Optical tweezer inducing cell membrane bending. (**C**) Optical trapping and placing of cells onto the micro-scaffold [75]. Copyright 2017, WILEY−VCH Verlag GmbH. (**D**) Schematic diagram of magnetic tweezer.

The optical tweezer (OT) method can be considered the most versatile single-molecule manipulation microscopy technique, and is commonly used to measure the rheological properties [64,65] and membrane tension of cells. In 1969, A. Ashkin first reported observing the acceleration and trapping of free-suspended particles using the radiation pressure of continuous visible laser beams [66]. Then, in 1986, the same group successfully trapped dielectric particles ranging from 25 nm to 10 μm using a single-beam gradient force trap [67]. The trapping force arises from the gradient force of the optical field and is strong enough to overcome scattering forces, forming a stable three-dimensional potential well. Dielectric particles can be trapped and moved like tweezers in this manner, marking the birth of optical tweezers (Figure 1B). Researchers can actively capture and manipulate particles, and the size of biological cells (typically 20–30 μm) perfectly matches the micromanipulation characteristics of OT (Figure 3A). At the beginning, researchers needed to incubate the particles, such as 1.5 μm silica beads that can be manipulated by optical tweezers, at different locations in the cells, such as: (i) attached to the membrane surface [68,69,70]; (ii) embedded in the cytoplasm [71,72]; (iii) targeted to specific proteins [73,74]. The optical micromanipulators can apply forces exceeding 100 pN to particles, while measuring the three-dimensional displacement of trapped particles with sub-nanometer precision and sub-millisecond time resolution. These capabilities make OT an ideal tool for measuring forces and movement.

One of the most important applications in the tissue engineering of OT is the measurement of tether forces at the single-cell level. The membrane tension can be estimated through the tether forces in cells (Figure 3B) [76]:

(3) $$T=F_{tether}^{2}/8B\pi^{2}$$

where T is the tether force measured using an optical tweezers system equipped with an infrared laser source and B is the bending stiffness of the membrane, defined as the force for a given curvature radius [77].

The tether force can be calculated using Hooke’s law:

(4) F=kΔx

where k is the stiffness of the optical trap and x is the displacement of the trapped particle by the optical tweezers.

Membrane tension is one of the most important physical criteria for assessing the cellular state in cell mechanics such as cell migration and spreading phenotypes [78,79], stem cell differentiation [80,81,82,83], and epithelial–mesenchymal transition [84,85]. Moreover, the high precision of OT enables their direct use in assembling the physical structures of complex cell microenvironments [86]. Kirkham et al. used holographic optical tweezers to directly assemble cellular microstructures [87]. Gullo et al. designed wireframe microscaffolds for the predetermined arrangement of cells by using OT (Figure 3C) [75]. Cells were optically captured and positioned on the microscaffolds, forming precisely arranged superstructures of multicellular hybrid organisms.

The principle and testing goals of magnetic tweezers is similar, but also involves manipulating beads to exert force on cells and observe their mechanical responses (Figure 3D) [16]. The magnetic tweezers method involves controlling the movement and rotation of magnetic particles using a magnetic field, allowing various stretching experiments at the microscopic scale [32]. MT can manipulate magnetic beads ranging in size from 0.5 to 5 μm, making them suitable for analyzing the mechanical properties of cells [88,89,90]. Moreover, by culturing cells with a defined dose of magnetic nanoparticles in a patterned manner on a micro-magnetic substrate, researchers can directly observe highly coordinated responses in the behavior of thousands of cells in a single experiment and achieve high-statistical-precision measurements of cortical tension [91]. Furthermore, MT can also be used to measure the linear and nonlinear viscoelastic behavior of cells and the ECM in a specific environment [92,93,94].

### 2.3. Traction Force Microscopy

**Figure 4:**
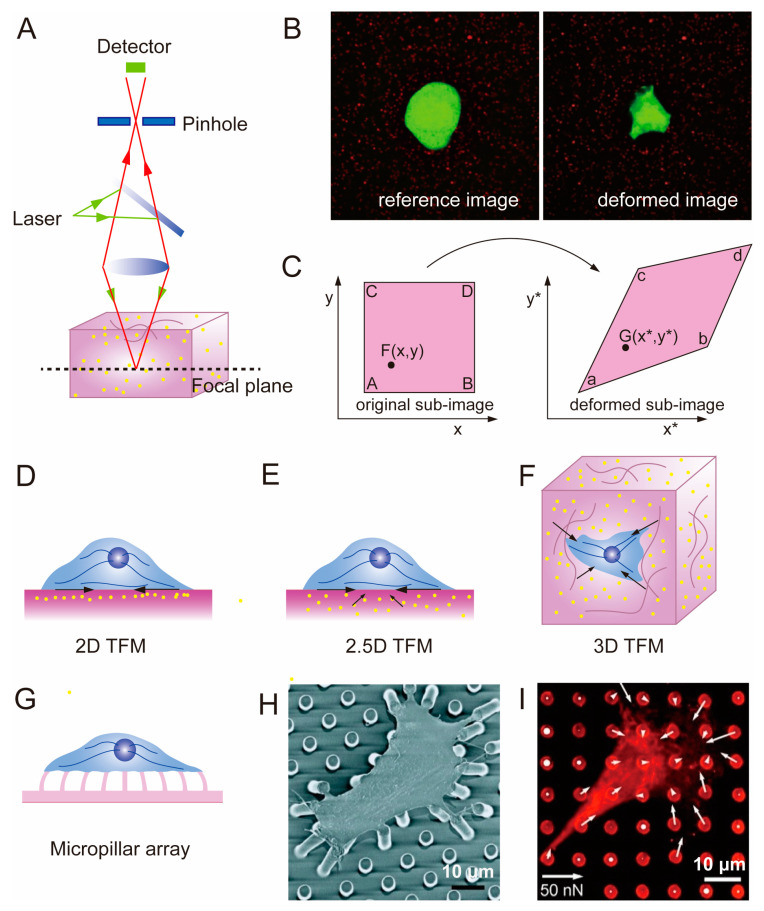
TFM based on deformable image processing and its application on different matrix. (**A**) Confocal microscopy can perform scanning of fluorescent microbeads within biological soft samples. (**B**) Two 3D confocal images were acquired through different fluorescence channels for green cells and red fluorescent beads, respectively [35]. Copyright 2016, Elsevier. (**C**) Basic concept of deformation mapping by DIC. (**D**–**F**) Schematic diagram of 2D TFM (**D**), 2.5D TFM (**E**), 3D TFM (**F**). The black arrows indicate the direction of traction forces exerted by cells on the substrate. (**G**) Diagram of micropillar array. (**H**,**I**) Smooth muscle cell attached to micropillars [108]. Copyright 2003, National Academy of Sciences. Scanning electron micrograph (**H**), confocal images (**I**). The white arrows refer to the force exerted by cells, calculated from the position of fibronectin on the tips of the posts.

TFM, invented by Oliver and others in the 1990s [95], is a microscopy technique used to study cell-generated traction forces in the ECM [96]. “Traction force” refers to the actual force (in dynes, 10^−5^ N) exerted by a whole cell or a part of it on its underlying substrate, while “traction” denotes the force density (in dyn/cm^2^) exerted by the cell at any specific point on the substrate. TFM combines microscopic observations of material deformation in the cell matrix with algorithms that convert optical signals into mechanical signals. TFM heavily relies on confocal microscopy, since all subsequent displacement field and stress field calculations are based on images captured by CLSM (Figure 4A). With the advancement of confocal technology, precise deformation images can be obtained, enabling more accurate and reliable quantitative detection of mechanical microenvironments at the cellular scale [97,98,99]. By culturing cells on or within an elastic substrate embedded with fluorescent microbeads, the displacement of the microbeads caused by ECM deformation mediated by cells can be observed. By tracking the changes in the positions of the microbeads and obtaining the displacement field of the elastic substrate based on digital image correlation (DIC) and digital volume correlation (DVC) algorithms, the corresponding cell-generated stress field can be calculated based on elastic constants such as the material’s elastic modulus and Poisson’s ratio [35,96,100,101]. The coordinate changes of fluorescent microbeads are obtained by comparing the “reference image” and the “deformed image” (Figure 4B) [32]. The reference image is the image of the fluorescent microbeads in the original sample without any material deformation, while the deformed image refers to the distorted form due to traction forces applied by cells. By comparing the two images and observing the bead displacement using confocal microscopy, a displacement field of the substrate can be visualized. Then, the stress field surrounding individual cell can be reconstructed based on the theory of elastic mechanics, using algorithms and modeling software such as ABAQUS [102], ANSYS [103,104], COMSOL [105,106,107], etc.

DIC and DVC algorithms are optical metrology methods based on digital image processing and numerical computation. They allow for obtaining displacement and strain fields by comparing digital images of the undeformed and deformed surfaces of a sample in two-dimensional space. The fundamental principle involves establishing correspondences between particle coordinates in the reference and deformed images, establishing a large-deformation function relationship based on the continuum mechanics theory (Figure 4C). DIC has extensive applications in mechanical testing at the microscale and nanoscale, as well as in the large deformation analysis of soft materials [109]. DVC is a 3D spatial algorithm developed based on DIC technology [110,111]. In TFM studies focusing on cells cultured on soft elastic substrates, the DVC algorithm is often used to determine the 3D displacement induced in the substrate by cellular activities [112]. The application of TFM in cells has transitioned from 2D and 2.5D to 3D. Typically, 2D TFM and 2.5D TFM are used when seeding cells on a thin substrate with tunable elasticity, whereas 3D TFM refers to embedding cells within a matrix [113]. In 2D TFM, only the lateral displacement is measured (Figure 4D), while in 2.5D TFM, both the lateral and axial components of the displacement are measured simultaneously [114] (Figure 4E). 3D TFM quantifies the induced stresses in three dimensions by embedding cells in an elastic materials (Figure 4F) [115].

Micropillar array is a discrete 2D TFM technique that is used to measure the forces generated by cells cultured on a 2D matrix (Figure 4G), which avoids the mathematical complexity of TFM. The spatially resolved cell forces can be calculated by measuring the deflection of individual pillars (Figure 1D) [116,117,118,119]. Micropillar array and 2D TFM share the common feature of quantifying displacement fields and retrieving stress fields based on markers on a 2D cell culture substrate (Figure 4H,I) [100]. The difference lies in the fact that the elastic micropillar array’s substrate material can only be silicon (Si) elastomeric polydimethylsiloxane (PDMS) [108]. Due to the strict requirements of the micropillar array regarding manufacturing materials, its application in the mechanical conduction of cells in tissue engineering scaffolds is limited. However, interesting findings can still be derived, such as the linear increase in traction forces exhibited by fibroblasts and epithelial cells on micropillar arrays with increasing stiffness [120].

### 2.4. Finite Element Analysis

Finite element analysis (FEA), as a purely theoretical computational approach, holds value in guiding experimental directions and providing reference for other experimental results, especially in chondrocyte studies [121,122,123]. Mow et al. first proposed a linear biphasic model for studying articular cartilage biomechanics [124]. This model incorporates both linear elastic solid material and non-viscous fluid material, with the fluid material being able to diffuse through the solid material. The observed viscoelastic behavior of cells and tissues in biological experiments can be explained by the momentum exchange between the two materials through friction [125]. J.Z. Wu et al. developed a model that simulated the mechanical behavior of articular cartilage and described the stress–strain and fluid pressure distributions in chondrocytes as time-dependent during cartilage deformation [126]. They assumed that the cell-matrix composite material can be mechanically equivalent to a homogeneous matrix, with both structures having the same elastic strain energy when exposed to a uniform stress field.

FEA can simulate different types of mechanical behaviors and loading conditions without direct contact or the intervention of cellular biological samples. However, the mechanical behavior of cells is a complex physiological process, and the simplifications made by FEA may result in differences between the calculated results and the actual situation. Alexopoulos et al. developed a finite element method that combined experimental and numerical approaches. They utilized a micropipette aspiration technique to obtain morphological changes in cells and used the obtained data as parameters for finite element modeling to calculate the mechanical properties of chondrocytes [123]. FEA can also be used to address the limitations of AFM, such as the nonaxisymmetry of the tip geometry and inclination angle of the cantilever relative to the cell surface [127].

## 3. Application of Advanced Mechanical Testing Technologies in Tissue Engineering

**Figure 5:**
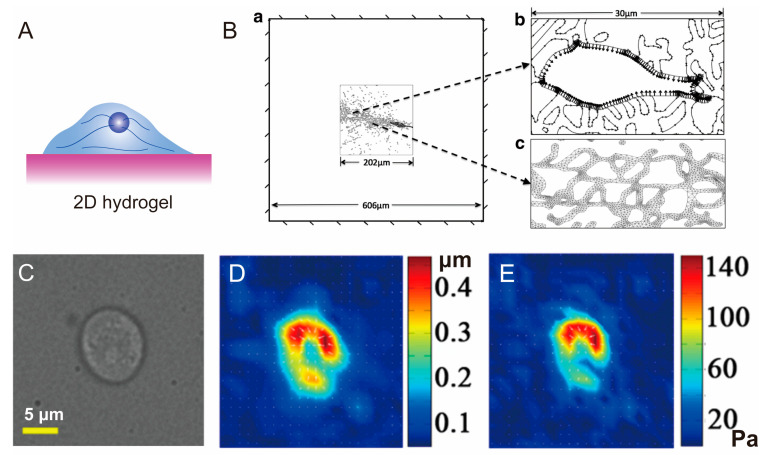
2D Hydrogel Applications of Advanced Mechanical Testing Technologies. (**A**) Illustration of 2D hydrogel for cell cultivation. (**B**) 2D solid-plane stress models with three subdomains [158], representing fibroblasts seeded on top of type-I collagen gels. Copyright 2013, Elsevier. (**a**) Fixed boundary conditions with the ROI surrounded by 2D hydrogel. (**b**) Uniform contractile stress simulating contracting cell. (**c**) A dense mesh used to compute stress distributions in the fibers. (**C**–**E**) Representative images of cell spreading on the PA gel [159]. Copyright 2018, American Association for the Advancement of Science. (**C**). Phase contrast. (**D**) Displacement map of the gel, generated from the lateral deformation of PA gels based on the coordinates of fluorescent microspheres. (**E**) Stress map obtained through inverse calculation.

By introducing advanced mechanical testing techniques, including AFM, OT, MT, TFM, and FEA, the mechanical microenvironment of a matrix and the mechanical properties of the cells themselves can be detected and analyzed. Based on these mechanical properties and the biological responses of cells, we can reveal the mechanotransduction mechanism to optimize the mechanical compatibility of scaffold materials, and even study the relationship between diseases and mechanics. Hydrogels [128,129,130,131] and porous scaffolds [132,133,134] are frequently used materials in tissue engineering scaffolds, all belonging to elastic matrices. For the mechanical detection of these elastic matrices, two basic assumptions are commonly adopted: (i) the materials used for cell culturing and measuring cell traction forces are both linear and isotropic [30,135,136]; (ii) compared to the size of the cells, the dimensions of the substrate are infinite, so the cells do not perceive the stiffness of the underlying rigid glassware [137,138], meaning that the deformations caused by cell traction forces are unaffected by the overall geometry and size of the substrate [96]. Figure 5 includes schematic diagrams of these matrices with different structural and experimental results obtained from the mechanical probing of these matrices.

### 3.1. Hydrogel for 2D Cell Cultivation

Hydrogels have garnered significant attention in the field of tissue engineering due to their versatile nature, including excellent biocompatibility and tunable mechanical properties. Hydrogels can be composed of natural materials such as hyaluronic acid [139,140,141], gelatin [142,143,144], collagen [139,145,146], and silk fibroin [147,148,149,150]. Through the establishment of covalent and non-covalent bonds, these materials form a three-dimensional network structure, transitioning from a solution to a gel state [151]. The mechanical properties of hydrogels can be precisely regulated through the molecular design of polymers, the manipulation of intermolecular crosslinking, and various processing and compounding techniques, such as double network hydrogels. Hydrogel scaffolds closely mimic the biomimetic extracellular matrix to realize cell–matrix interaction, providing cells with a biophysical and biochemical microenvironment that facilitates cell activities [152]. Emerging research has demonstrated that the mechanical properties of hydrogels, including stiffness [153,154], stress relaxation [155], dynamic stiffening, and softening [156,157], play a crucial role in modulating the adhesion, spreading, proliferation, and differentiation of stem cells. Gaining a deeper understanding of how to detect and transmit mechanical signals to cells, as well as the subsequent cellular responses, is pivotal for optimizing the preparation parameters of hydrogel materials and facilitating the development of effective tissue engineering scaffold materials. Cell cultivation in 2D hydrogel means planting cells on the surface of hydrogel (Figure 5A).

By performing 2D geometric modeling and the meshing of cells planted on the surface of hydrogels to conduct FEA, Xiaoyue Ma et al. revealed that stresses generated by a centripetally contracting cell boundary are concentrated in the relatively stiff ECM fibers and are propagated further in a fibrous matrix as compared to homogeneous linear elastic or strain-hardening materials [158]. They chose to simulate the cell contraction of one cell by imposing centripetal stress on its membrane (Figure 5B). This finite element modeling approach can better elucidate the role of ECM fibers in stress transfer, providing deeper insights into the selection of biomaterials for tissue engineering applications.

Hydrogels are well-suited for embedding fluorescent beads for traction force microscopy as they can transition from a precursor solution state to a gel state. Polyacrylamide (PA) and soft polydimethylsiloxane (PDMS) are commonly used materials for the preparation of hydrogels for studying 2D-cultured cells because they are transparent and their stiffness can be easily tuned. Moreover, prior to cell seeding, hydrogels are frequently coated with extracellular matrix (ECM) proteins to enhance cell adhesion. This coating process optimizes the interaction between the hydrogel surface and the seeded cells, facilitating their attachment and spreading within the hydrogel matrix. In 2D hydrogels, fluorescent beads can be placed on the surface of the hydrogel or embedded within the hydrogel [100,159,160] (Figure 4D,E). For instance, in a study by Wang et al., in 2018, fluorescent beads were anchored on the surface of a PA gel to track the traction forces exerted by B cells against antigen substrates [159] (Figure 5C–E). Computer-assisted tracking of the fluorescent beads using DIC algorithms allows for the measurement of gel deformation. The research findings demonstrated that the traction forces exerted by B cells were primarily induced by BCR antigen recognition in response to antigen substrates, while basal traction forces were minimal in the absence of antigen substrates.

By employing advanced mechanical testing techniques like TFM, researchers have been able to investigate the regulatory mechanisms underlying cell phenotype conversion in response to mechanical heterogeneity. In a study conducted by Feng Lin et al., the researchers revealed that mechanical heterogeneity alone is capable of spontaneously inducing a mesenchymal-like phenotype, highlighting the critical role of biophysical cues in triggering phenotypic switching [160]. Furthermore, they employed TFM to measure the traction forces generated by MCF7 cell monolayers on micropatterns and observed substantial variations in traction forces between the peripheral and central regions of the cells. Additionally, by utilizing cantilever-based nanoindentation, the researchers quantitatively assessed the Young’s modulus of MCF7 cell monolayers on the micropatterns and observed a decrease in cell stiffness following the process of epithelial–mesenchymal transition [160]. These findings provide valuable insights into the relationship between mechanical cues, cell phenotype conversion, and changes in cellular mechanical properties. 

Cellular behavior is regulated by mechanotransduction signaling pathways [161]. The stiffness and surface topography of the extracellular matrix can synergistically influence the recognition of topographical features by cells. Cells cultured on 2D substrate can be examined using AFM. By fitting typical AFM force–distance curves using the Hertz model, the Young’s modulus of the cells can be calculated. Researchers have found that U2OS cells cultured on flat quartz (72 GPa) exhibit higher stiffness and plasma membrane tension compared to cells cultured on PA hydrogel (1 kPa) [162]. However, in a distinct approach, researchers investigated the behavior of cells cultured on a quartz substrate featuring quartz nanopillars generated through electron beam lithography. Despite the substrate possessing a comparable stiffness to that of flat quartz substrates, the Young’s modulus and membrane tension of the cells cultivated on the nanopillars detected by AFM exhibited similar results to the cells cultured on soft hydrogels. This means that the topographical morphology of the nanopillars counteracted the influence of a rigid substrate on the cells.

Yali Yang et al. utilized a modified MT device to achieve precise force application on polyacrylamide hydrogels [163]. By attaching 45 μm magnetic beads to the gel surface and applying magnetic forces of approximately ~10 pN to the hydrogel surface, they measured the response of cellular microtubule networks to localized mechanical perturbations. It was inferred that local compression and bending forces in the hydrogel regulate the mechanics of the cellular cytoskeleton at the micron scale, rather than tensile restoring forces. All these studies have confirmed the significance of microscopic advanced mechanical detection technologies for the analysis of mechanotransduction mechanisms and cell–matrix interaction by providing more information. 

### 3.2. Hydrogel for 3D Cell Cultivation

**Figure 6:**
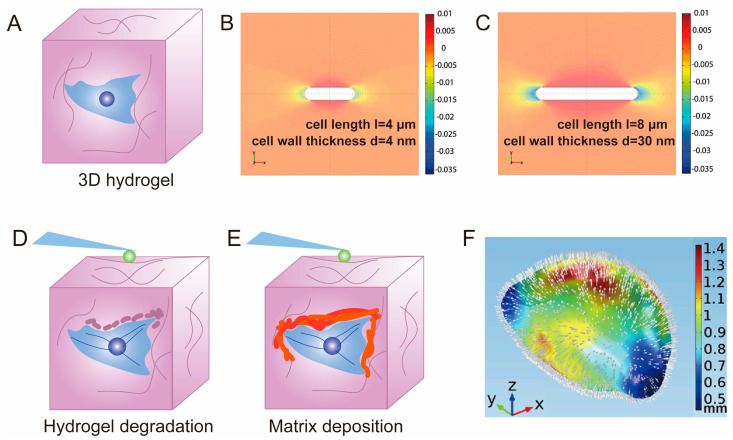
3D Hydrogel Applications of Advanced Mechanical Testing Technologies. (**A**) Illustration of a cell−laden 3D hydrogel. (**B**,**C**) Estimation of cellular mechanical properties based on 3D simulations of growth [172]. Copyright 2012, Blackwell Publishing. A rod-shaped bacterium with a radius of 0.5 mm, cell wall thickness of 4 nm (**B**) or 30 nm (**C**), and cell initial lengths of 4 μm (**B**) or 8 μm (**C**), embedded in a gel with E = 56 kPa. (**D**) Schematic diagram of AFM measurement of cells embedded in 3D hydrogel and partially degraded hydrogel around cells; (**E**) stem cells undergo secretory sedimentation in the pericellular matrix; (**F**) displacement field at the cell−gel interface. A typical 3D distribution of cell growth-induced displacement at 24 h in a gel. White arrows indicate the relative magnitude and direction of the displacement [35]. Copyright 2016, Elsevier.

An additional notable feature of hydrogel scaffold materials is their ability to encapsulate cells for 3D cultivation [152,164,165,166]. Most cells in native tissues exist within a 3D mechanical microenvironment, making the mechanical interaction between cells and their surrounding 3D microenvironment a topic of great interest in biomechanical research. Alongside the aforementioned natural biomaterials, certain synthetic materials with excellent biocompatibility, such as polyethylene glycol (PEG), can provide a suitable 3D microenvironment for encapsulated cells through mild cross-linking reactions [167]. In contrast to cell cultivation on 2D surfaces, 3D cell cultivation involves the encapsulation of living cells within materials (Figure 6A). Importantly, cells exhibit distinct behavioral characteristics in 2D and 3D environments [168]. For instance, cells seeded on rigid substrates tend to exhibit a greater spreading compared to cells on softer materials [169]. However, cells encapsulated in 3D hydrogels demonstrate enhanced spreading capabilities within hydrogel with a soft stiffness [2]. Moreover, the encapsulation state and spreading state of cells within 3D hydrogels exhibit distinct characteristics in terms of cell morphology, cytoskeleton assembly, and local adhesion structures [170]. The spatial arrangement of cells significantly influences cellular mechanotransmission processes. For example, Benito et al. successfully achieved a spindle-shaped cytoskeleton, the behavior of which resembled that of cells migrating within a native 3D extracellular matrix, by sandwiching fibroblasts between two layers of PA hydrogel [171]. This sandwich-like structure reduces the number and size of local focal adhesions, facilitates adhesion anchorage at the front and back of cells, and reduces the cell migration speed.

Hannah H. Tuson et al. provided a FEA technique for the mechanical modeling of bacterial cells encapsulated in 3D hydrogels [172]. They modeled the cell envelope as a cylindrical shell with hemispherical endcaps (Figure 6B,C), with deformation energy arising from bending, stretching, and swelling forces. The hydrogel was represented as an assembly of interconnected small volumes with an isotropic Young’s modulus. This report validated their prediction that, under the same hydrogel stiffness, longer cells should experience more growth inhibition, and also provided reference values for establishing finite element models of 3D cells encapsulated in hydrogels.

Although AFM is primarily used for studying the surface structure and properties of solid materials, as well as detecting weak atomic-level interactions between the surface of a test sample and microforce sensing elements, it can also be utilized to assess the spatial mechanical characteristics of hydrogel with cell encapsulation. Eric M. Darling et al. quantified the microscale elastic modulus of their region of interest using AFM and stiffness mapping techniques [173]. They fabricated a template resembling the PCM using soft lithography, which consisted of a stiff PA gel filling the gel center and a surrounding soft PA gel. The stiffness distribution in hydrogels exhibits spatial variability around cells and in extracellular matrices away from cells as observed by AFM [174]. To measure the Young’s modulus E at the cellular scale, they randomly selected multiple locations on the hydrogel surface for force measurement. It was found that human mesenchymal stem cells (hMSCs) can rapidly modify the stiffness of the PCM by secreting proteins, making it stiffer than the bulk matrix, or softening the pericellular matrix by inducing degradation, thereby rapidly altering the local mechanical microenvironment of the cells (Figure 6D,E). Geraldine M. Jowett used AFM-based indentation to map the stiffness variations mediated by type-1 innate lymphoid cells (ILC1) encapsulated in a 3D hydrogel, revealing that cells actively regulate their mechanical microenvironment by driving matrix softening and stiffening [59]. Notably, the results detected by AFM only partially reflect the differences in the elastic modulus between the pericellular matrix and the volumetric hydrogel, and cannot directly represent the modulus of the pericellular matrix [48].

The application of TFM to 3D hydrogels necessitates the careful consideration of multi-dimensional data gathering. Unlike 2D TFMs, which are limited to measuring displacements within the plane, 3D TFMs enable the measurement of displacements in all three dimensions. This is accomplished by employing the DVC algorithm [136,175], which facilitates the acquisition of a 3D strain field. Subsequently, this strain field can be inverted to calculate the corresponding 3D stress field, providing valuable insights into the mechanical interactions between cells and their surrounding matrix within the 3D hydrogel environment. This innovative approach helps in understanding the complicated mechanical behaviors and forces that are present in 3D cellular microenvironments.

Huang Jianyong et al. proposed an innovative approach using hydrogels as sensors to quantify cellular forces in a 3D environment [35]. The method begins with the mechanical characterization of cell-encapsulated methylcellulose (MA) hydrogels to determine their elastic modulus (E) and Poisson’s ratio (μ) by AFM. By fitting the force–depth curves, the stiffness of the hydrogel is derived, assuming that the hydrogel is a homogeneous, incompressible Neo-Hooker material. To quantify the Poisson’s ratio, the hydrogel was compressed, and reference and deformed images were obtained by tracking fluorescent beads that were previously encapsulated in the gel. Using DVC technology, the average normal strain (εx, εy, εz) was calculated by measuring the displacement of the fluorescent beads, thereby obtaining the Poisson’s ratio. This provides the material mechanics parameters that are required for subsequent modeling. Next, the cells were encapsulated in a hydrogel to simulate cell growth forces. The cell morphology was obtained by scanning with confocal microscopy and subjecting the cells to digital processing. A 3D model reconstruction was performed using COMSOL. To obtain reference and deformed images, the cells underwent hyperosmotic treatment. Similarly, the deformation field was obtained using the DVC algorithm in MATLAB, and the stress field was reconstructed using COMSOL (Figure 6F), allowing for a comprehensive analysis of the mechanical forces and interactions between the encapsulated cells and the surrounding hydrogel matrix.

Unlike on 2D hydrogel surfaces, when cells are encapsulated in 3D hydrogels, their growth pattern involves gel invasion rather than crawling. With cell–matrix interaction, cellular forces can trigger signaling pathways that affect cell proliferation and differentiation. Wesley R. Legant’s work showed that the greatest strain on hydrogels around cells occurs in the protruding pseudopodia [115]. It was observed that the highest stresses during spreading generally occurred in regions that were the focal point of adhesion, where stresses were significantly higher than in non-spreading cells. The cell–matrix interaction forces measured by these advanced mechanical testing technologies can help to explain the mechanotransduction mechanism in cell spreading and differentiation [176,177].

### 3.3. Porous Scaffold

**Figure 7:**
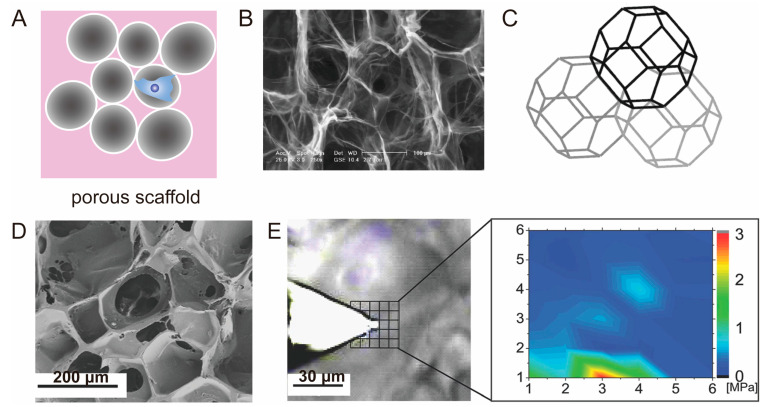
Porous scaffold Applications of Advanced Mechanical Testing Technologies. (**A**) Illustration of a porous scaffold. (**B**,**C**) Collagen-GAG scaffold [34]. Copyright 2010, Elsevier. (**B**) scanning electron micrograph, (**C**) tetrakaidecahedral model extracted from the porous scaffold. (**D**,**E**) AFM measurement of porous scaffold [62]. Copyright 2015, WILEY−VCH Verlag GmbH. (**D**) Scanning electron microscopy image of the porous scaffold. (**E**) Optical microscopy-guided positioning of the AFM tip for the indentation experiments on this ArcGel. The grid shows the corresponding indentation area. Enlarged is the corresponding indentation map giving the local Young’s modulus.

Porous scaffolds featuring interconnected pore structures have gained significant applications in the field of tissue engineering (Figure 7A), particularly in the field of bone and cartilage tissue engineering and regeneration [134]. The interconnected pore structure within these scaffolds can provide a beneficial space for cell migration and proliferation, while facilitating efficient nutrient transportation. Various techniques can be employed to create the desired interconnected pore structure, such as employing sacrificial materials like ice templates to obtain interconnected pores [178,179], or utilizing 3D printing methods to fabricate regular porous structures [180,181]. By manipulating factors such as the material composition, pore size, and porosity, it is possible to obtain porous scaffold materials with varying mechanical properties. The macroscopic mechanical properties are typically assessed using a universal tensile machine. When directly implanted into the body, the scaffold’s macroscopic mechanical properties must meet the mechanical demands of the damaged tissue site. Prior to implantation, some scaffold materials are incubated in vitro, allowing for the enhancement of the scaffold’s mechanical properties over a specific period. Subsequently, the scaffold can be implanted into the body, providing an optimal environment for tissue regeneration.

K.A. Corin proposed a finite element modeling approach in their study to assess cell contractility within highly porous scaffolds of varying pore sizes and cell densities [34]. The porous scaffold was simplified as a truncated tetrahedra with varying structural lengths (Figure 7B,C). To calculate the contractile force exerted by fibroblasts, the researchers employed finite element analysis and applied forces to individual tetrahedrons. They assumed point loads that were eccentrically applied on the surface of each pillar end of the tetrahedra to simulate cell attachment and contraction. The finite element model demonstrated that the buckling force of each pore in the porous scaffold decreases with increasing cell density, while the buckling force of each cell increases with the square of the pore size. These finite element simulations will help to understand the mechanical interaction of cells and porous scaffolds and can provide a theoretical basis for the optimization of the mechanical properties of porous scaffolds.

The porosity and pore size of the scaffold can be tailored during the fabrication process to meet the specific requirements of cell growth and tissue regeneration. For instance, the pore size can directly influence the growth of cartilage and bone tissue, while the mechanical properties of the pore wall significantly impact cell adhesion and differentiation on the surface of pores [62]. The mechanical microenvironment of cells in porous scaffolds plays a crucial role in regulating cell fate; thus, there is a focus on detecting the forces generated by cells or the mechanical microenvironment sensed by cells, such as the elastic modulus. For example, to determine the local microscale mechanical properties of ArcGels, which can guide cell homing and induce osteogenic differentiation, A.T. Neffe et al. developed an approach which combines optical microscopy and AFM (Figure 7D,E) [62]. In brief, to avoid any protruding parts of the porous scaffold from coming into contact with the AFM cantilever, they used optical microscopy to identify regions with heights smaller than the AFM probe height (3 μm), and then determined their locations by measuring force–distance curves at individual points on the scaffold and calculating the Young’s modulus.

In a study conducted by Damien Robert et al., MT were utilized to accurately measure the apparent viscosity of the internal channels and the stiffness of the pore walls within porous scaffolds [182]. The researchers employed a specific method wherein stem cells were labeled with magnetic nanoparticles, allowing them to function as probes in order to assess the mechanical microenvironment surrounding the cells. Accurate quantification of the micromechanical properties of porous scaffolds holds paramount importance in scaffold design and stem cell differentiation. It is worth noting that the initial mechanical properties of the porous scaffold alone do not solely determine the fate of cells. Over time, the mechanical properties of the PCM can undergo significant changes due to the degradation of the matrix or the deposition of macromolecules. These alterations in mechanical properties can be precisely detected using MT. Consequently, monitoring these changes becomes critical in understanding the dynamic nature of the microenvironment and its influence on cellular behavior.

## 4. Conclusions and Future Perspectives

The mechanical signals of tissue engineering scaffolds can regulate cell behaviors such as adhesion, spreading, migration, and differentiation. Since the complex mechanical interaction between cells and extracellular matrices exists on a microscopic scale, mechanical testing technology at the cellular level is gradually being developed and applied. Herein, the mechanisms and applications of current techniques used to detect forces exerted by cells in the matrix and the mechanical properties of the cellular microenvironment are summarized and discussed. Firstly, the principles of basic microscale mechanical detection techniques, including AFM, OT, MT, TFM, and FEM are elucidated. AFM, OT, and MT can measure parameters such as the modulus by monitoring the deflection or displacement of the instrument probe, while TFM uses the matrix as a force sensor carrier and inversely calculates the displacement field corresponding to the matrix deformation to obtain the distribution of cellular stresses and strains. Subsequently, an exposition is provided on the application of these techniques to tissue engineering scaffolds, encompassing hydrogels and porous scaffolds. The suitability of various microscale mechanical sensing techniques for different types of scaffolds is influenced by factors such as dimensional aspects, material properties, and mechanical characteristics. We offer a comprehensive evaluation of the advantages and limitations associated with these factors (Table 1), with the objective of providing inspiration and guidance to researchers working in the field of cellular mechanical response within the extracellular matrix.

Simultaneously or nearly simultaneously detecting the material mechanical properties of the cell microenvironment and the stress at the cell–matrix interface at the same location remains a challenge in microscale mechanical testing techniques at the cellular level. When detecting cellular stresses, it is necessary to directly determine the elastic modulus and Poisson’s ratio of the matrix; otherwise, it is not possible to calculate the stress from the deformation. For example, Huang Jianyong et al. obtained the mechanical properties of elastic hydrogels using 3D TFM before cell encapsulation, and after encapsulating cells within the hydrogel, they directly used the mechanical parameters of the hydrogel without cells to calculate the stress in cell-laden hydrogel [35]. However, during cell growth, cells actively remodel their matrix environment through the secretion of extracellular proteins, which can either stiffen the surrounding matrix through deposition or soften it through degradation. Therefore, the mechanical properties of the matrix and the stress are required to be detected at the same time. In this regard, a potential solution is provided by the controlled magnetic microrobotic probe developed by Erfan Mohagheghian et al. [189]. This method utilizes a magnetic field to induce rigid rotation of the probe to measure the shear modulus of cell clusters, and quantifies the deformation caused by the surrounding cells to detect stress, thereby enabling the quantification of both the modulus and three-dimensional traction forces at the cellular level using the same probe. However, due to the inherent size limitation of the robotic probe, reaching up to 50 μm, this magnetic probe can only be embedded in cell clusters such as tumor communities or embryos, allowing for the mechanical characterization of cell clusters but not individual cells. Therefore, it has limited significance in exploring the mechanical response at the cell–matrix interface between tissue engineering scaffolds and single cells. Nevertheless, the concept still contributes valuable insights and serves as a reference for addressing the aforementioned challenges.

In addition, cellular responses are supported by subcellular structures, and the investigation of the mechanical properties of these structures can further advance the research in the field of cell mechanics [190,191,192]. Many structures and organelles within cells are composed of proteins, which participate in cell signaling and regulation, enabling cells to perceive and respond to mechanical stimuli from the matrix [193,194,195], thereby regulating gene expression, cell proliferation, differentiation, and apoptosis. Although the biomechanical signaling at the cell–matrix interface has been studied, there is still a need for further exploration of the mechanical behavior of mechanoregulatory proteins [196]. Currently, researchers have utilized AFM to apply external forces to individual PIEZO channel proteins to study their structural unfolding, folding, and elastic properties [197]. Further research is needed to investigate the signaling mechanisms of proteins under mechanical responses within live cells [198]. Therefore, there is a need to continually improve the existing instruments and develop new technologies for micromechanical testing. This will result in a more comprehensive and in-depth understanding of the behavior of cells in response to physical cues, enabling us to address future challenges regarding biophysical problems in the life sciences.

## Figures and Tables

**Table 1:** Summary of the techniques for mechanical microenvironment detection.

Technique	Test Parameters	Target Parameters	Application	Advantages	Limitations
AFM	Cantilever deflection	Modulus of cell or substrates	2D hydrogel [162,183] 3D hydrogel [59,173,174] Porous scaffold [62]	Force measurements in real-time	Surface contact
OT	Optical beads displacement	Modulus of cell or substrates	3D hydrogel [184,185]	High resolution	Heating; photodamage
MT	Magnetic beads displacement	Modulus of cell or substrates	2D hydrogel [163] 3D hydrogel [186] Porous scaffold [182]	No photodamage	Limited sensitivity
TFM	Displacement of fluorescent beads	Strain, stress	2D hydrogel [159,160] 3D hydrogel [35,115]	Non-invasively. Wide applicability	High sensitivity to noise. Dependency on software
FEM	/	Strain, stress	2D hydrogel [158,183,187] 3D hydrogel [172,188] Porous scaffold [34]	Qualitative modeling	Low accuracy

## Data Availability

The datasets generated and/or analyzed during the current study are available from the corresponding author upon reasonable request.
